# Supplementary material for: Conserved assembly architecture of the essential herpesvirus packaging accessory factor
Source: PLoS Pathog. 2026 Sep 10;22(9):e1014540. doi: 10.1371/journal.ppat.1014540 (PMC13561365; doi:10.1371/journal.ppat.1014540)

Supporting Information - Original images for blots and gels

Fig. 1B

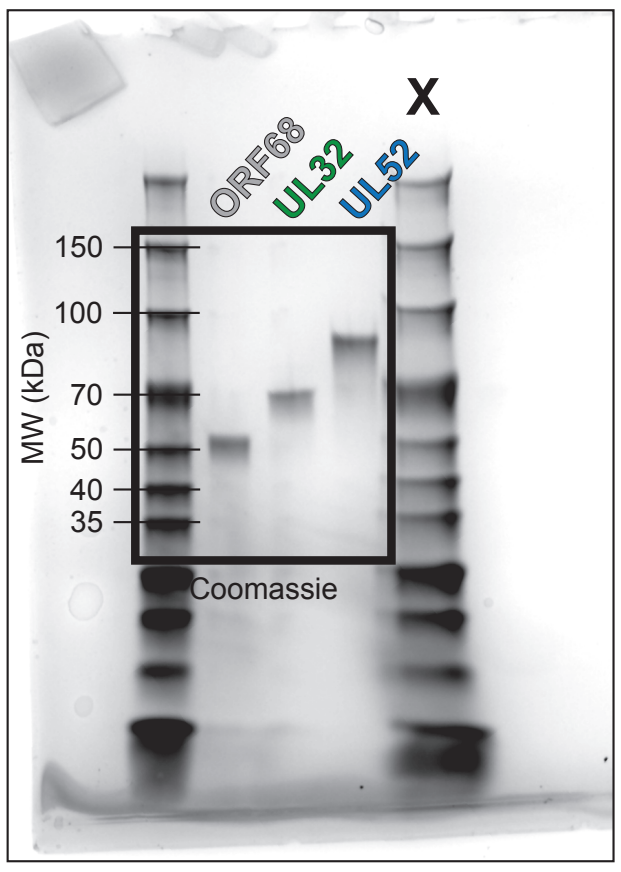

Fig. 5C

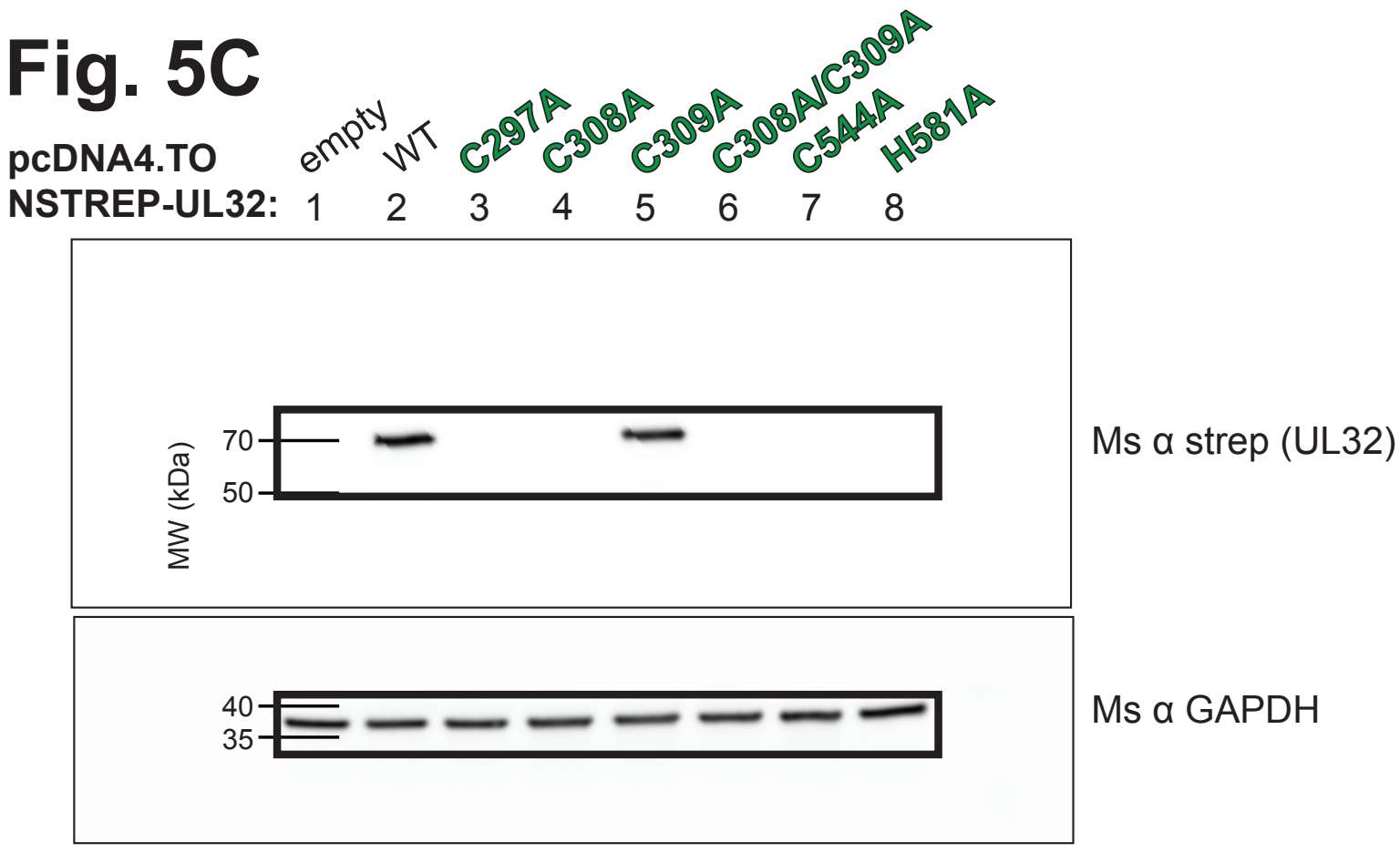

Supplementary Fig. S4A

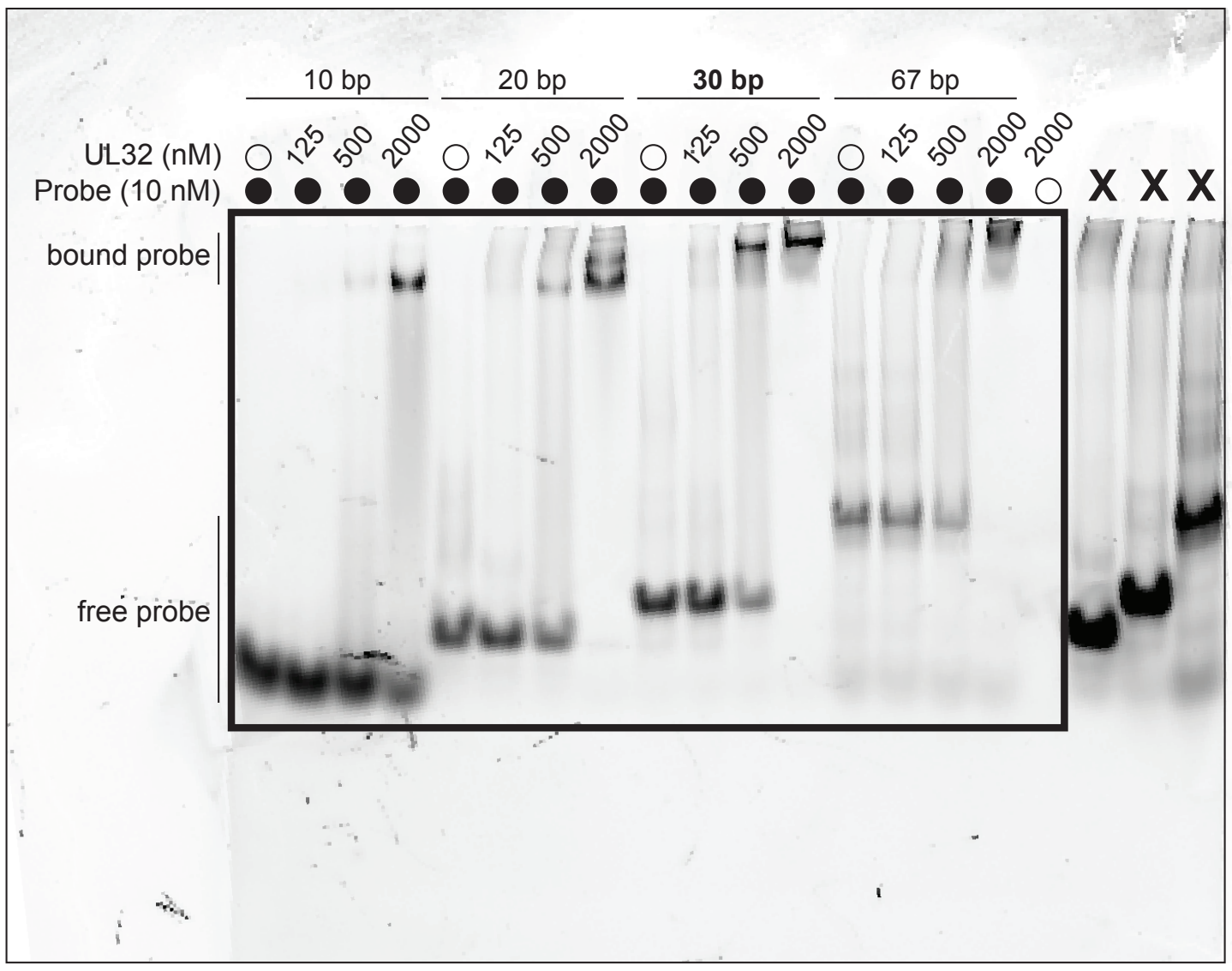

Supplementary Fig. S4B

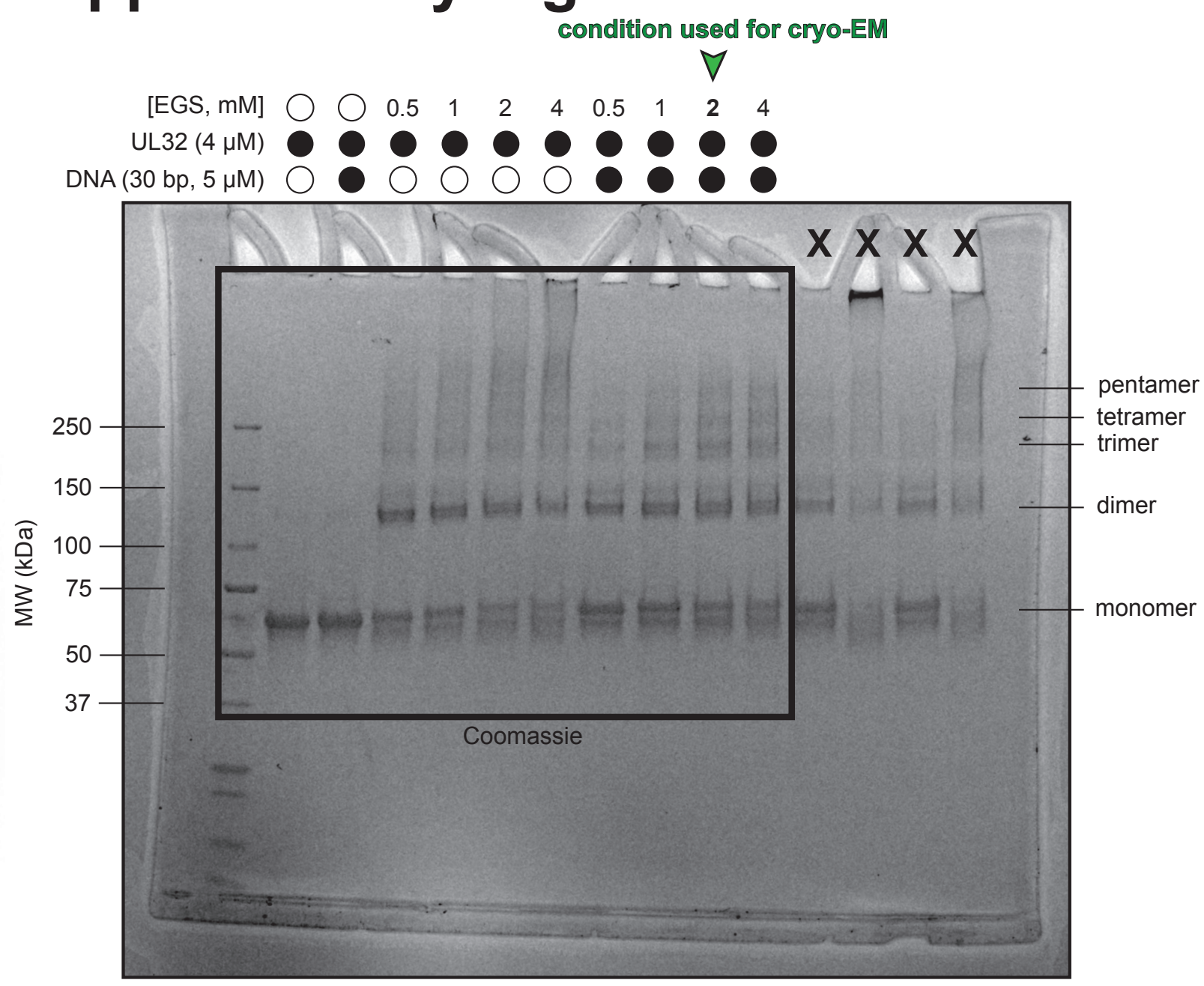

Supplementary Fig. S4C

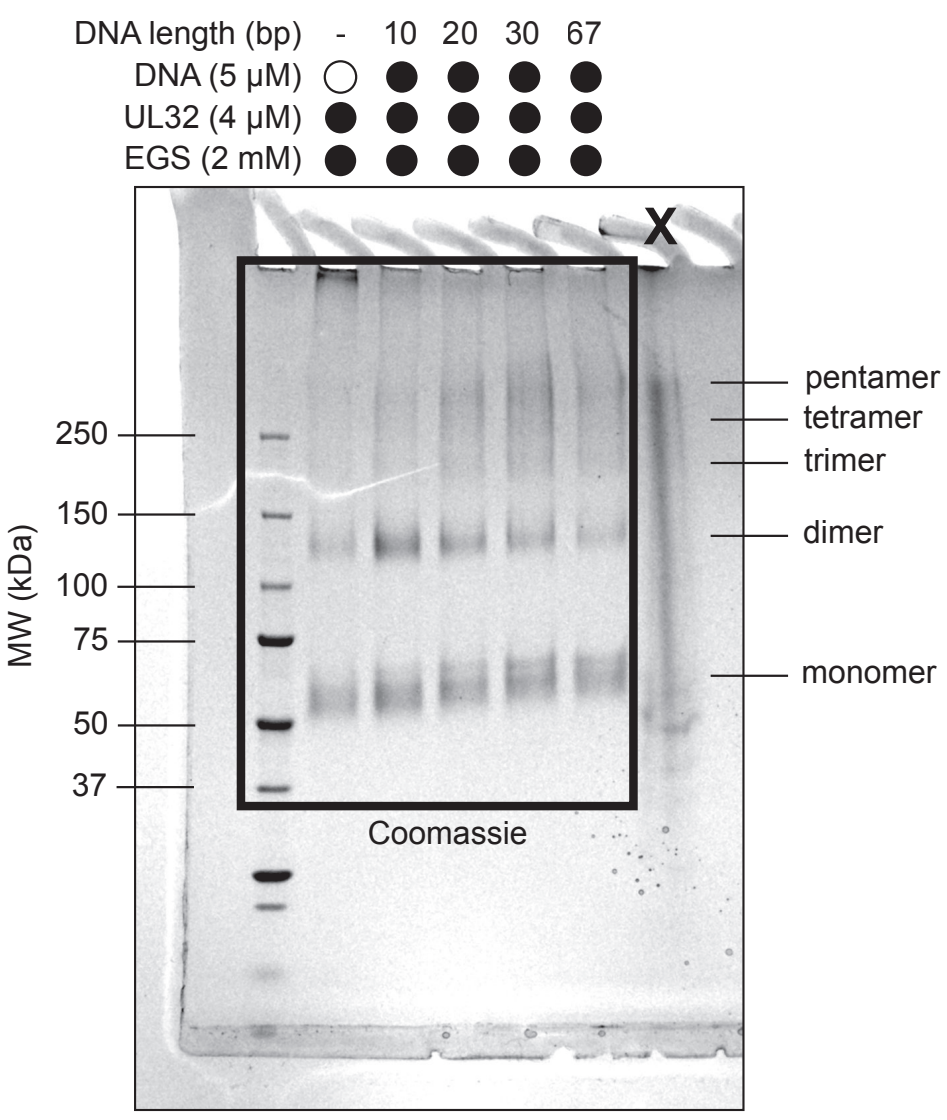

Supplementary Fig. S7E

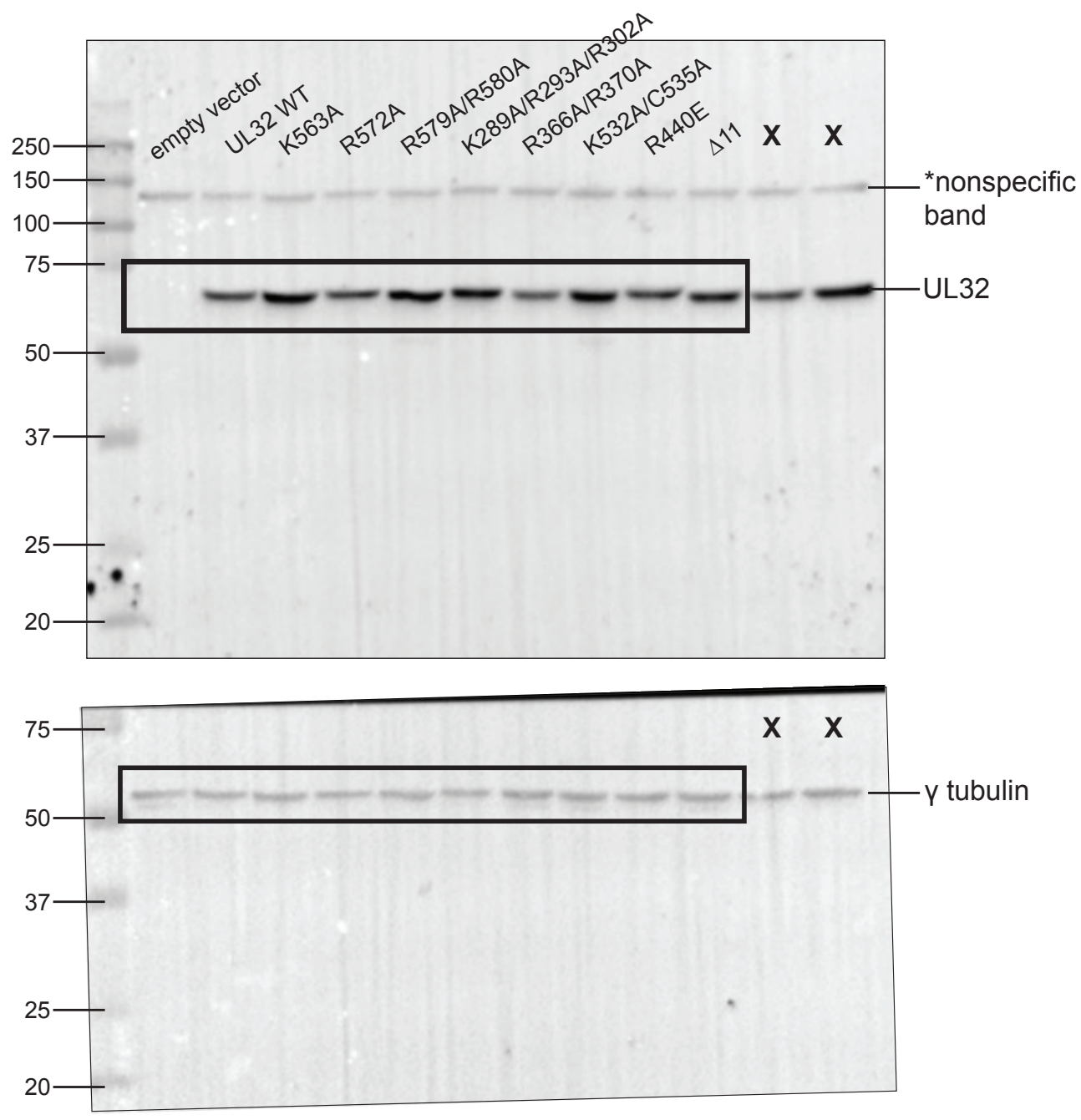

Supplement: Supplementary file 1 [file ppat.1014540.s010.pdf]
